# Investigating SARS-CoV-2 RNA in five municipal wastewater treatment plants, hospital wastewater and wastewater collection networks during the COVID-19 pandemic in Ardabil Province, Iran

**DOI:** 10.1007/s13201-022-01773-6

**Published:** 2022-10-18

**Authors:** Abdollah Dargahi, Mehdi Vosoughi, Ali Normohammadi, Anoshirvan Sedigh, Helia Gholizadeh, Hadi Sadeghi, Chiman Karami, Farhad Jeddi

**Affiliations:** 1 Social Determinants of Health Research Center, Ardabil University of Medical Sciences, Ardabil, Iran; 2 Department of Environmental Health Engineering, School of Health, Ardabil University of Medical Sciences, Ardabil, Iran; 3 Department of Microbiology, School of Medicine, Ardabil University of Medical Sciences, Ardabil, Iran; 4 Department of Genetics and Pathology, School of Medicine, Ardabil University of Medical Sciences, Ardabil, Iran

**Keywords:** SARS-CoV-2, Municipal wastewater, Coronavirus, Wastewater treatment plant, Collection network, Hospital wastewater

## Abstract

Since 2019, the outbreak of coronavirus with acute respiratory symptoms has caused an epidemic worldwide. Transmission of the disease through respiratory droplets was announced as the main mode of transmission in 2020. But in this study, we discussed the method of indirect transmission of the virus through sewage. In this study, effluents related to urban and hospital wastewater treatment plants in 5 regions of Ardabil Province (northwest of Iran) were investigated. In this research, 120 samples were kept in pre-test conditions (temperature -20 degrees Celsius). To identify the viral genome, special primer and chain reaction probe targeting ORF1ab and N (nucleoprotein gene) genes were used. Out of a total of 120 samples, a total of 3 samples were positive. Wastewater epidemiology (WBE) can be considered as a cost-effective method in the diagnosis and prediction of pathogenic agents. And be considered an effective method for decision-making in order to protect the health of citizens.

## Introduction

In December 2019, a number of pneumonia cases were identified in Hubei Province, China And then this disease was reported in different countries And finally, the health organization declared this disease as an epidemic (Alygizakis et al. 2021; Chekol and Melesse 2020). Although some virus risk factors are mild, some viruses are associated with acute diagnostic symptoms (Amoah et al. 2020; Lai et al. 2020a). The presence of viral agents in the surrounding environment, including surface water sources, is unavoidable. Therefore, investigating the existence of these factors and their genetic sequences can be effective in terms of managerial and health decisions (Asghar et al. 2014; Han and He 2021). Some diseases such as polio and coronaviruses have caused many casualties and deaths at different times throughout history (Jiehao et al. 2020; Alahdal et al. 2021). In recent years, with the spread of coronaviruses, clinical symptoms have been widely recorded, according to various reports, including respiratory, digestive and fatigue complications (Michael-Kordatou et al. 2020; Vosoughi et al. 2021). With various epidemics over time, there is a concern of consuming water with these pathogens. Water sources can be polluted by various factors such as improper wastewater collection processes, unauthorized activities, lack of monitoring of the performance of wastewater collection systems, especially in developing countries, hospital effluents, etc., and endanger the public health of citizens and people who work in water and sewage treatment units (Mallapaty 2020; Lahrich et al. 2021; Carducci et al. 2020).

In previous studies, the presence of SARS-CoV-2 and RNA in discharged wastewaters has been mentioned, which shows the need to pay attention to health systems (Waggoner et al. 2020; Hillary et al. 2020). Although droplets and aerosols in the respiratory air of a sick person were introduced as the main way of transmission by the World Health Organization in 2020, it is also important to check the indirect ways of transmission (Wilson et al. 2022; Manoj et al. 2020). According to the types of virus transmission methods and reports of the presence of this virus in human feces samples, it is important to check these studies have been done in this regard in different countries, which shows the importance of the issue (Lodder and Roda Husman 2020). In recent studies, the results indicate that RNA attributed to SARS-CoV-2 is present in the stool samples of sick people and people carrying the disease of Covid-19 (Gao et al. 2020; Holshue et al. 2020). Epidemiology investigation based on wastewater is a new method to investigate the situation of virus outbreak (Xagoraraki and O’Brien 2020; Sen-Crowe et al. 2020). Based on the studies conducted, it seems that this method can be used as a suitable way to detect the prevalence and to issue a warning before the identification of pathogenic agents and it can be effective for maintaining the public health of the society (Hillary et al. 2020; Hellmér et al. 2014). Considering the wide spread of this disease all over the world, Iran was not excluded.

and many people were infected so that at one point in Iran's time, it ranked first in the world in terms of the rate of infection. And on the other hand, the analysis of the results of the experiments obtained from the wastewater was very important from the point of view of various researchers.

And the statistics showed that the presence of these viruses for a maximum of 3 days in untreated sewage can have various effects and threaten human health by infiltrating surface and underground waters (Jiehao et al. 2020; Medema et al. 2020; Naddeo and Liu 2020). It has been conducted with various studies in this field in countries such as European countries, Australia, America, etc. (Lodder and Roda Husman 2020; Medema et al. 2020; Wu et al. 2020). But in this research, we are going to investigate SARS-CoV-2 in the wastewater collection networks, wastewater treatment plants and hospitals wastewater in the cities of Sarein, Meshkinshahr, Germi, Bilehsavar and Parsabad.

## Materials and method

### Study area

This study was conducted in Ardabil Province, one of the largest provinces in the north-west of Iran. According to the Statistical Centre of Iran, the total of Ardabil Province inhabitants was estimated to be 1.3 million in 2019, wherein 48% were women. Administratively, the province is divided into 10 cities, and in total, there are 13 public hospitals and 5 private hospitals in the province.

### Specimen collection, storage, and transfer

**Fig. 1:**
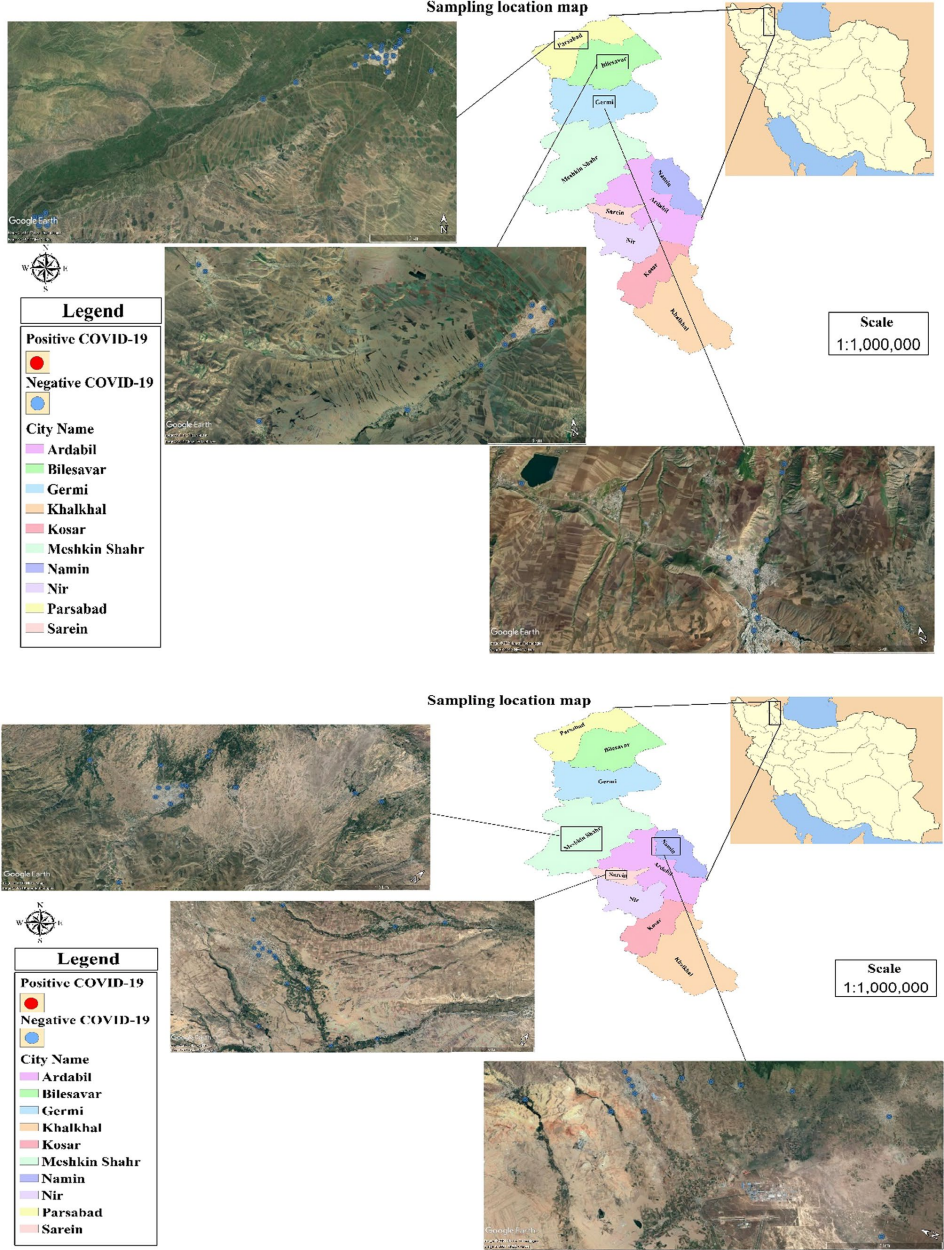
Position of different sampling points in this study (Parsabad, Aslanduz, Bilehsavar, Germi, Meshkinshahr, Namin, Sarein cities)

In this project, wastewater samples have been collected from the effluents and effluents of hospital and urban wastewater treatment plants from the wastewaters of the cities of Ardabil Province, including Aslanduz, Sareyn, Meshginshahr, Garmi, Bileh Savar, Parsabad and Namin. Ardabil Province is located in the northwest of Iran and is considered as one of the provinces with critical conditions in terms of the spread of coronavirus (which is shown in Fig. 1).

Sampling of the desired areas is according to Fig. 1. And the sampling officials acted according to health protocols to collect the samples and used health and safety personal protective equipment (Sen-Crowe et al. 2020). The volume of each collected sample was 250 ml that the conditions of sampling and storage of each sample were done according to the standard technique. In determining the number of samples, attention was paid to several factors, which include: the number of existing hospitals and treatment plants, examining similar studies and modeling from these studies. And based on these factors, 120 samples were finally taken from the mentioned areas. Sampling was done from a specific point at different times from 10 a.m. to 2 p.m. and combining them together (combined sampling). The samples were stored in the laboratory at − 20 degrees to inactivate bacterial activity to analyze the samples at the right time. A 24-h sample of raw wastewater was taken from the wastewater and pasteurized at 60 °C for 90 min to inactivate the virus according to the hygienic protocol for sewage sampling.

### RNA extraction method

Then, a filter (Millipore Sigma) with 0.45 µm pores was used to remove bacterial cells. 100–200 ml of the sample (Universal 320R) was centrifuged at 4750 g for 30 min.

These cases were done to concentrate the samples separated from the sewage and finally, the supernatant was carefully removed without disturbing.

Supernatants were then centrifuged at 3500 g for 15 min along with the centrifugal filter with a cut-off of 10 kDa. Centrifugation was performed for 2 min at a speed of 1000 rpm of course, during this time, the container containing the sample was upside down on top of the sample filter cup. RNA extraction kit (viral nucleic acid kit with high purity) was used to collect the sample. And we took 250 µL of the concentrated sample with a pipette.

Bacteriophage ø6 was used as external control in order to determine the effectiveness of RNA extraction. Appropriate primers and probes were designed for N and RDRP genes to perform real-time PCR.

To identify the virus in the initial screening stage, attention was paid to various factors, which include: concentration of reagents, checking the temperature cycle and the number of reproduction cycles. The specific primer and probe real-time reverse transcriptase–polymerase chain reaction (Real-Time PCR) targeting ORF1ab and N genes (Nucleoprotein gene) were applied to detect viral genomes of the SARS-CoV-2 virus in the wastewater samples.

Applied Biosystems™ Real-Time PCR System 7500 with software v2.0.5 was used to run Real-Time PCR. Appropriate concentrations of the synthesis reaction for Real-time PCR MasterMix are as follows: H_2_O (RNAse free) 1.1 μL, 2 × Reaction mix 12.5 μL, MgSO_4_ (50 mM) 0.4 μL, BSA (1 mg/mL) 1 μL, Primer RdRP_SARSr-F and 2019-nCoV_N F (10 µM stock solution) 1.5 μl, Primer RdRP_SARSr-R and 2019-nCoV_NR (10 µM stock solution) 2 μL, Probe RdRP_SARSr-P and 2019-nCoV_N P (10 µM stock solution) 0.5 μ, SSIII/Taq EnzymeMix 1 μL, total reaction mix 20 μL, template RNA, add 5 μL, total volume 25 μL. Thermal Cycler: 55 °C 10′ 94 °C 3′ 94 °C 15″ 58 °C 30″, 45 ×.

### Positive and negative control

Positive Control2019-nCoV-qPCR was used to monitor the correct performance of qRT-PCR in each step. Negative Control 2019-nCoVqPCR was used to monitor whether there was any contamination for the rRT-PCR course in each detection run. An internal control in the extraction step was used by Wuhan Coronavirus N-gene kit (TIB Molbiol, Berlin, and Germany) to authorize the extraction and PCR amplification procedure.

### Running real-time PCR and data analysis

To identify the SARS-CoV-2 virus, the microtube containing the extracted genome was subjected to a real thermal cycler along with other reaction components and then the Real-time PCR results were interpreted by the operator based on the data analysis criteria.

## Results and discussion

Table 1 shows the primers and probes used in this research and the target genes. Table 2 shows the samples taken from 5 areas of Sareyn, Meshkinshahr, Garmi, Bileh Savar and Parsabad. In each of the areas, two types of samples were taken from the incoming sewage and the outgoing sewage and in total, out of 10 samples collected, 3 samples are positive.

**Table 1:** Primers and probes used in this study

Organisms	Target gene	Sequence (5′–3′)	Cycling parameters
SARS-CoV-2	Probe & Primer ORF1a/b	FACAGGTGGAACCTCATCAGGAGATGC-BBQ F-GTGARATGGTCATGTGTGGCGG R-CARATGTTAAASACACTATTAGCATA	55 °C 10′ 94 °C 3′ 94 °C 15″ 58 °C 30″ 45 ×
	Primer &Probe N gene	F-AAATTTTGGGGACCAGGAAC R-TGGCAGCTGTGTAGGTCAA PFAM-ATGTCGCGCATTGGCATGGA-BHQ	55 °C 10′ 94 °C 3′ 94 °C 15″ 58 °C 30″ 45 ×

**Table 2:** Identification of Covid 19 virus in wastewater treatment plants

Wastewater treatment plant	Coronavirus status		
	Results	Ct-ORF1ab gene	Ct-N gene
Sarein			
Inlet wastewater	Negative	–	–
Outlet wastewater	Negative	–	–
Meshkinshahr			
Inlet wastewater	Positive	36.26	34.48
Outlet wastewater	Negative	–	–
Germi			
Inlet wastewater	Positive	35.81	33.56
Outlet wastewater	Negative		
Bilehsavar			
Inlet wastewater	Negative	–	–
Outlet wastewater	Negative	–	–
Parsabad			
Inlet wastewater	Positive	33.41	29.78
Outlet wastewater	Negative	–	–

All three positive samples are related to incoming sewage. The status of the coronavirus in these three samples is also reported according to the table. Table 3 shows the identification of the Covid-19 virus in the wastewater of hospitals and health centers in these 5 regions. Which shows the data collected from 12 hospitals that reported negative results in this review. The results of the identification of the covid-19 virus in the sewage collection network in the regions (Parsabad, Aslanduz, Bileh Savar, Garmi and Namin) are shown in Table 4. Out of a total of 66 samples collected from the sewage collection network in the mentioned areas were declared negative for the presence of coronavirus. Table 5 shows identification of the Covid-19 virus in the sewage collection network (Meshkinshahr and Sareyn). The results of the identification of the Covid-19 virus in the sewage collection network (Meshkinshahr and Sareyn) are reported in Table 5, out of a total of 32 tested samples, all negative cases were recorded. As discussed earlier in relation to the various methods of transmission of the coronavirus, we know that respiratory droplets are the main way of transmission but transmission through contaminated sewage is also significant and since 1.8 billion people in the world use contaminated water as drinking water, this issue is important. And checking the amount of sewage pollution can be effective in monitoring the spread of disease (Bhowmick et al. 2020; Lai et al. 2020b). In fact, sewage is a complex collection of a wide variety of viruses (Martínez-Puchol et al. 2020). Performing tests based on the genetic identification of the coronavirus can be effective in identifying other pathogens; however, the risk of sewage contamination is possible with animal coronaviruses in addition to human coronaviruses (Martínez-Puchol et al. 2021). The presence of coronavirus in the feces of patients even after 5 weeks after the respiratory samples was negative reveals the fact that monitoring this case can be effective. Even when the disease conditions are better, it can help in predicting the future situation (Wölfel et al. 2020; Lesté-Lasserre 2020). Sewage monitoring can be considered as a sensitive tool on virus circulation in the environment (Yuan et al. 2021; Elsamadony et al. 2021). It is important to investigate such indirect factors in the spread of the disease. In particular in developing countries, because in these countries there is a high probability of contamination of fresh water in drainage (Ali et al. 2020). In a review of wastewater-based epidemiology (WBE), evidence suggests that the persistence of the coronavirus in municipal and hospital wastewater is largely dependent on temperature. And practically the most stability of the virus happens at low temperature (Mandal et al. 2020; Kumar et al. 2021). In general, the stability of coronaviruses in water is higher than that of uncoated intestinal viruses; therefore, it is considered important to monitor the wastewater of the coronavirus in terms of environmental health (Bonanno Ferraro et al. 2021). The results of this study show that one of the cost-effective ways to monitor the conditions of Corona is to conduct relevant tests on sewage. Because it is possible to identify infected people and even carriers and take the necessary measures before the peak of the disease outbreak among the major problems and limitations in this study is the lack of facilities such as ultracentrifuge, DNA sequencing pointed out.

**Table 3:** Identification of Covid 19 virus in hospitals and health centers wastewater

Cities	Hospitals	Results	Ct-ORF1ab gene	Ct-N gene
Parsabad and Aslanduz	Imam Khomeini	Negative	–	–
	Shohada	Negative	–	–
	Aras	Negative	–	–
	Aslanduz Health Center	Negative	–	–
	Parsabad Health Center	Negative	–	–
Germi	Velayat	Negative	–	–
	Germi Health Center	Negative	–	–
Bilehsavar	Imam Khomeini	Negative	–	–
	Bilehsavar Health Center	Negative	–	–
Meshkinshahr	Imam Khomeini	Negative	–	–
	Meshkinshahr Health Center	Negative	–	–
Namin	Namin Health Center	Negative	–	–

**Table 4:** Identification of Covid 19 virus in wastewater collection network (Parsabad, Aslanduz, Bilehsavar, Germi, and Namin)

City	Sample code	Results	Ct-ORF1ab gene	Ct-N gene	City	Sample code	Results	Ct-ORF1ab gene	Ct-N gene
Parsabad and Aslanduz	1	Negative	–	–	Bilehsavar	10	Negative	–	–
	2	Negative	–	–		11	Negative	–	–
	3	Negative	–	–		12	Negative	–	–
	4	Negative	–	–		13	Negative	–	–
	5	Negative	–	–		14	Negative	–	–
	6	Negative	–	–	Germi	1	Negative	–	–
	7	Negative	–	–		2	Negative	–	–
	8	Negative	–	–		3	Negative	–	–
	9	Negative	–	–		4	Negative	–	–
	10	Negative	–	–		5	Negative	–	–
	11	Negative	–	–		6	Negative	–	–
	12	Negative	–	–		7	Negative	–	–
	13	Negative	–	–		8	Negative	–	–
	14	Negative	–	–		9	Negative	–	–
	15	Negative	–	–		10	Negative	–	–
	16	Negative	–	–		11	Negative	–	–
	17	Negative	–	–		12	Negative	–	–
	18	Negative	–	–		13	Negative	–	–
	19	Negative	–	–	Namin	1	Negative	–	–
	20	Negative	–	–		2	Negative	–	–
	21	Negative	–	–		3	Negative	–	–
	22	Negative	–	–		4	Negative	–	–
	23	Negative	–	–		5	Negative	–	–
	24	Negative	–	–		6	Negative	–	–
Bilehsavar	1	Negative	–	–		7	Negative	–	–
	2	Negative	–	–		8	Negative	–	–
	3	Negative	–	–		9	Negative	–	–
	4	Negative	–	–		10	Negative	–	–
	5	Negative	–	–		11	Negative	–	–
	6	Negative	–	–		12	Negative	–	–
	7	Negative	–	–		13	Negative	–	–
	8	Negative	–	–		14	Negative	–	–
	9	Negative	–	–		15	Negative	–	–

**Table 5:** Identification of Covid 19 virus in wastewater collection network (Meshkinshahr and Sarein)

City	Sample code	Results	Ct-ORF1ab gene	Ct-N gene	City	Sample code	Results	Ct-ORF1ab gene	Ct-N gene
Meshkinshahr	1	Negative	–	–	Sarein	1	Negative	–	–
	2	Negative	–	–		2	Negative	–	–
	3	Negative	–	–		3	Negative	–	–
	4	Negative	–	–		4	Negative	–	–
	5	Negative	–	–		5	Negative	–	–
	6	Negative	–	–		6	Negative	–	–
	7	Negative	–	–		7	Negative	–	–
	8	Negative	–	–		8	Negative	–	–
	9	Negative	–	–		9	Negative	–	–
	10	Negative	–	–		10	Negative	–	–
	11	Negative	–	–		11	Negative	–	–
	12	Negative	–	–		12	Negative	–	–
	13	Negative	–	–		13	Negative	–	–
	14	Negative	–	–		14	Negative	–	–
	15	Negative	–	–		15	Negative	–	–
	16	Negative	–	–		16	Negative	–	–

## Conclusion

Our results show that it is possible to carry out the mentioned tests in wastewater. And the use of these results can be suitable for decision makers in the field of health to take preventive measures before the peak of the disease occurs and in general, it can be said that WBE is a suitable and economical method to detect the spread of the virus in the people of a region.

## Data Availability

The dataset and analyzed during the current study are available from the corresponding authors on realistic demand.
